# Surface plasmon resonance microscopy identifies glycan heterogeneity in pancreatic cancer cells that influences mucin-4 binding interactions

**DOI:** 10.1371/journal.pone.0304154

**Published:** 2024-05-22

**Authors:** Jesús S. Aguilar Díaz de león, Miyuki Thirumurty, Nguyen Ly

**Affiliations:** Biosensing Instrument Inc., Tempe, Arizona, United States of America; University of Mississippi, UNITED STATES

## Abstract

Membrane proteins are the main targets of therapeutic drugs and most of them are glycosylated. Glycans play pivotal roles in several biological processes, and glycosylation changes are a well-established hallmark of several types of cancer, including pancreatic cancer, that contribute to tumor growth. Mucin-4 (MUC-4) is a membrane glycoprotein which is associated with pancreatic cancer and metastasis, and it has been targeted as a promising vaccine candidate. In this study, Surface Plasmon Resonance Microscopy (SPRM) was implemented to study complex influences of the native N-glycan cellular environment on binding interactions to the MUC-4 receptor as this is currently the only commercially available label-free technique with high enough sensitivity and resolution to measure binding kinetics and heterogeneity on single cells. Such unique capability enables for a more accurate understanding of the “true” binding interactions on human cancer cells without disrupting the native environment of the target MUC-4 receptor. Removal of N-linked glycans in pancreatic cancer cells using PNGase F exposed heterogeneity in Concanavalin (Con A) binding by revealing three new binding populations with higher affinities than the glycosylated control cells. Anti-MUC-4 binding interactions of enzymatically N-linked deglycosylated pancreatic cancer cells produced a 25x faster association and 37x higher affinity relative to the glycosylated control cells. Lastly, four interaction modes were observed for Helix Pomatia Agglutinin (HPA) binding to the glycosylated control cells, but shifted and increased in activity upon removal of N-linked glycans. These results identified predominant interaction modes of glycan and MUC-4 in pancreatic cancer cells, the kinetics of their binding interactions were quantified, and the influence of N-linked glycans in MUC-4 binding interactions was revealed.

## Introduction

Membrane proteins play critical functions in cell biology, ranging from cell signaling, protein transport, and translocation [1]. Roughly 60% of all membrane proteins are the targets of therapeutic drugs [2–4]. However, to effectively understand the binding kinetics of new or potential therapeutic drugs, it is crucial to study binding in the context of the cell surface that contains a complex array of carbohydrate structures (glycans) associated with the membrane proteome, which are often overlooked. Most of the membrane proteins carry some form of glycan structure [5], and they play pivotal roles in protein folding, stability, cell communication, cell signaling, cell growth, protein structure and function [6], as well as antibody binding interactions [7, 8]. There are two main types of glycans in membrane glycoproteins: O-linked and N-linked glycans [9]. Aberrant N-linked and O-linked glycosylation is a hallmark of several types of cancer [10], and they are known to facilitate tumor development [11]. Increased expression of branched N-linked glycans on membrane glycoproteins and aberrant O-linked glycosylation on mucins are common features of pancreatic cancer (PC) cells [12] and many other types of cancer [12–24]. Consequently, profiling of N- and O-linked glycosylation is critical for the discovery of new diagnostics and cancer biomarkers, and to understand the function of tumorigenic membrane glycoproteins within the complex glycosylated cancer cellular environment.

Mucin-4 (MUC-4) is a heavily glycosylated membrane glycoprotein with O-glycans primarily contributing to its bulky structure in the extracellular region and some N-glycosylation sites that are part of the transmembrane region [25, 26]. MUC-4 is a novel tumor antigen that significantly contributes to PC development, which is absent in the normal pancreas, making it a highly attractive candidate for immunotherapy and vaccine development [18, 19, 21, 22, 27–30]. Additionally, the aberrant glycosylation within and around MUC-4 has shown to contribute to tumor growth [31–34]. Therefore, understanding the influence of aberrant glycosylation on the binding capability of MUC-4 in the native microenvironment is crucial to test the effectiveness of new drugs.

Several lines of evidence have shown that N-linked glycans on membrane glycoproteins can influence antibody binding interactions [7, 8, 35–38]. For instance, Lee et al. and Hu et al. have shown that removal of aberrant N-linked glycosylation in human cancer cells increases the affinity of the tumorigenic membrane glycoprotein PDL-1 [35–37], an important tumor antigen that is targeted for cancer therapy [35]. A later study has shown that N-linked glycosylation can alter receptor affinity by shielding specific regions in CD44 protein [39]. Other studies have shown that on immune cells, complex type N-linked glycosylation plays a critical role in modulating membrane FcγR affinity towards therapeutic antibodies [7, 8, 38, 40–44], rendering important implications in cancer immunotherapy [38].

More specifically, aberrant N-linked glycosylation is known to influence Herceptin binding to the oncoprotein HER2 in breast cancer cells [45]. Additionally, MUC-4 interacts with HER2, which has seven N-glycosylation sites in its extracellular domain and could be a potential hindrance to MUC-4 binding interactions [46–49]. However, to date, the influence of cell surface N-linked glycosylation on MUC-4 binding interaction kinetics with ligands remains unexplored.

Variations in binding interactions resulting from lectin or antibody polyvalency, different antigen glycoforms (branched and unbranched N-linked and O-linked glycans, glycosylated or deglycosylated), and receptor accessibility, are all collectively referred here as “heterogeneity”. The capability to determine binding heterogeneity as well as real-time binding kinetics simultaneously is a unique feature of surface plasmon resonance microscopy (SPRM). SPRM is a novel, highly sensitive technique that is gaining wide popularity for its ability to measure label-free kinetic binding interactions on whole single cells without disrupting the native environment of therapeutic targets [49–53]. This approach allows for a more accurate understanding of the “true” binding interactions of new or potential therapeutic drugs.

In this study, we profiled N- and O-linked glycan binding interactions in pancreatic cells using Concanavalin A (Con A) and Helix pomatia agglutinin (HPA) lectins, respectively, to evaluate their binding interactions and heterogeneity before and after partial removal of N-linked glycans using PNGase F enzyme [54]. We also examined the role of N-linked glycosylation, which is extraneous to the MUC-4 receptor, in MUC-4 binding interactions. To achieve this, we demonstrate the use of SPRM technology to study the influence of aberrant cell surface N-linked glycosylation on Con A, HPA, and MUC-4 antibody binding kinetics and heterogeneity on PC cells, which is not possible with traditional approaches that require membrane protein isolation and purification for the study of binding kinetics. Enzymatic N-linked deglycosylation of pancreatic cancer cells allowed testing of the hypothesis that aberrant N-linked glycosylation surrounding MUC-4 plays a major role in hindering its binding interactions.

## Materials and methods

### Materials and reagents

A SPRm 200 instrument, cell chamber kits (104–00228), and Au film sensors (106–00614) from Biosensing Instrument Inc. were utilized. Collagen from human placenta, Bornstein and Traub Type IV, powder, BioReagent, suitable for cell culture (catalog# C5533-5MG) was purchased from Millipore Sigma. Helix pomatia agglutinin lectin (HPA) (catalog# L11271), Concavalin A (Con A) (catalog# C11252), 4% paraformaldehyde (PFA) (catalog# J61899), and Hanks’ balanced salt solution (HBSS) (catalog# 14025076) were purchased from Thermo Fisher Scientific. Human MUC-4 Alexa Fluor® 488-conjugated Antibody (catalog# FAB8195G-100UG). Trypsin-EDTA solution, 1X (catalog# 30–2101) was purchased from ATCC. PNGase F (catalog# P0709S) was purchased from New England Biolabs (NEB).

### Cell culture

BxPC3 cell line (ATCC) was kindly provided by Dr. Shaopeng Wang’s lab of the Biodesign Institute at Arizona State University. BxPC3 cells were cultured in Eagle’s Minimum Essential Medium (EMEM) (ATCC cat# 30–2003) supplemented with 10% FBS. For subculturing, cells were allowed to reach 70–80% confluency. Cells were detached using a 0.05% trypsin-EDTA solution (ATCC). All cells were maintained in an incubator at 37°C with 5% CO_2_. For BI sensors chips, about 15K cells were seeded in BI sensor chips and allowed to grow for 72 hours at 37°C. Cells were then fixed with 4% PFA for 10 minutes, washed three times with HBSS, and immediately analyzed by SPRM. For every experiment, cell viability was determined with trypan blue using TC20 cell counter (BioRad).

### Cell preparation in sensor chips

To promote cell adhesion to the surface of the sensor chip (polymer well with Au chip), the sensor was filled with 500 μl of a 50 μg/ml collagen IV solution and incubated overnight at 4°C. Then, excess collagen IV solution was removed, and the sensor chip was rinsed with PBS buffer 3 times. Sensor chip was exposed to UV light for 10 minutes for sterilization. A total number of 15,000 BxPC3 cells at 99% cell viability were added to the sensor chip containing 400 μl of EMEM media supplemented with 10% FBS. Chip was then incubated at 37°C with 5% CO_2_. After 72 hours incubation, cells were washed with HBSS buffer 3 times to remove any FBS media. Cells were then fixed for 10 minutes with 4% PFA, washed three times in HBSS buffer, followed by SPRM analysis. For all enzymatic deglycosylation experiments, PNGase F enzyme at 2,000 units/ml was added directly to the fixed cells in sensor chip and incubated for 24 hours at 37°C. Then the cells were washed with PBS, followed by SPRM analysis.

### Surface plasmon resonance microscopy

**Fig 1:**
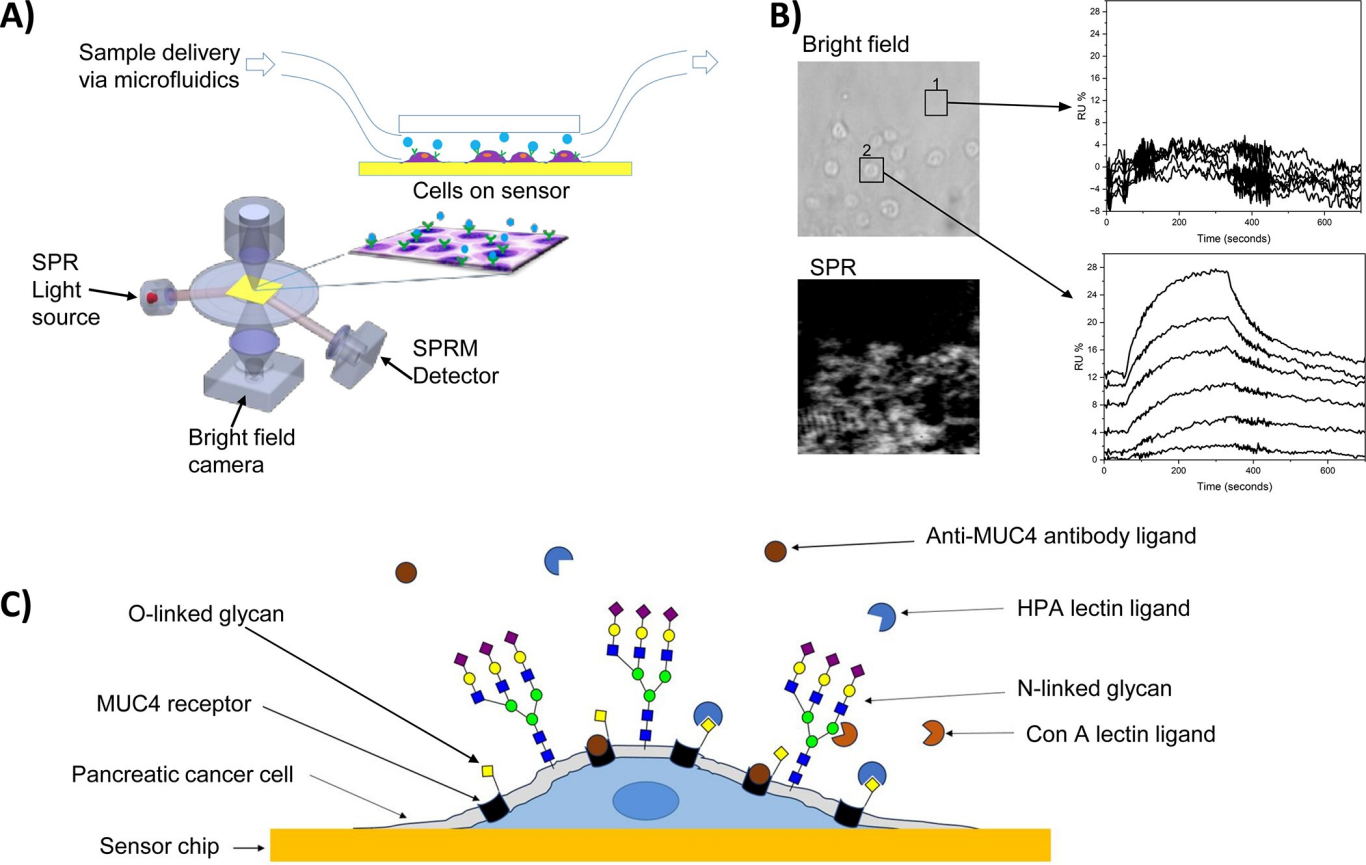
SPRM Principle and illustration of pancreatic cancer cell binding kinetics. **A**) Schematic showing simultaneous SPR detection and bright field visualization of individual cells. A transparent flow cell facilitates the optical imaging and precise delivery of sample solutions. **B**) bright field and SPR image of pancreatic cancer cells and samples of derived unfitted sensorgrams from bare area and single cell area, from which hundreds of sensorgrams from hundreds of cells on several different regions on the cell can be collected simultaneously. Black squares indicate sensorgram is derived from 1) bare area and 2) response from a single cell area. **C**) Adherent cell on sensor surface model for profiling of N- and O-linked glycan kinetics with Con A and HPA, respectively, and antibody kinetics with anti-MUC4 monoclonal antibody.

The SPRm 200 (surface plasmon resonance microscope model 200 from Biosensing Instrument Inc., Tempe, Arizona), which has an inverted microscope for simultaneous bright field imaging and SPR microscopy with CCD camera (600 μm × 450 μm area) [4, 50–52] was used in this study with BxPC3 adherent pancreatic cancer cells (**Fig 1**). The running buffer was 1X PBS with a flow rate of 100 μL/min. The cells in the sensor chip were then exposed to serial injections of HPA lectin 1/3 dilution solutions (0.102, 0.307, 0.923, 2.77, 8.33, 25, and 75 nM), Con A lectin 1/3 dilution solutions (0.102, 0.307, 0.923, 2.77, 8.33, 25, and 75 nM), and anti-MUC-4 antibody ½ dilution solutions (0.390, 0.781, 1.56, 3.12, 6.25, 12.5, 25 nM).

### Fluorescence assay

Cells were seeded at 15K cells/ml in sensor chip. After culturing for 72 hours at 37°C and 5% CO_2_, the wells were rinsed with PBS and the cells were fixed using 4% paraformaldehyde for 10 minutes. Then, 3% BSA was added to block the non-specific adsorption sites and incubated for 1 hr at 4°C. Alexa fluor 488 Con A lectin was added to the cells in the sensor chip at 75 nM and incubated for 5 minutes, and fluorescence was immediately recorded.

### Data fitting and analysis

The SPRM kinetic analysis results of all ROIs were aggregated for statistical analysis. Histograms of the kinetic parameters were fitted with Gaussian distributions to extract the mean and 95% confidence interval of the cell population [4, 50–52]. ImageSPR software (Biosensing Instrument Inc., Tempe Az) was used to generate the sensorgrams, fitting, and statistical analysis of binding interactions and heterogeneity. The association rate constant (*k*_*a*_), the dissociation rate constant (*k*_*d*_), and the equilibrium dissociation constant (KD) were extracted through fitting using a 1:1 or 1:2 kinetic binding model. To determine the most appropriate binding model, every interaction study was initially fit with a 1:1 kinetic interaction model, which determines the most localized and predominant mode of interaction. In the case where another influence on the interaction is observed such that the 1:1 error of fit is too high, a 1:2 kinetic interaction model is instead applied to compensate for secondary influences. If the error of fit remains above the acceptable threshold, then the response is deemed non-specific or does not fit classic kinetic binding interaction models and is filtered out. Additionally, by then varying both the sample concentration and measurement region, heterogeneity of the interaction can be explored and may also lead to the discovery of additional interaction modes. The histogram data obtained from the ImageSPR software were graphed and fitted using Origin 2023b graphing software. Image J was used to analyze Con A fluorescence data. Each fluorescence cell intensity was normalized to background fluorescence, and then plotted in GraphPad.

## Results

### Glycan and antibody kinetics evaluation on whole cells

**Fig 2:**
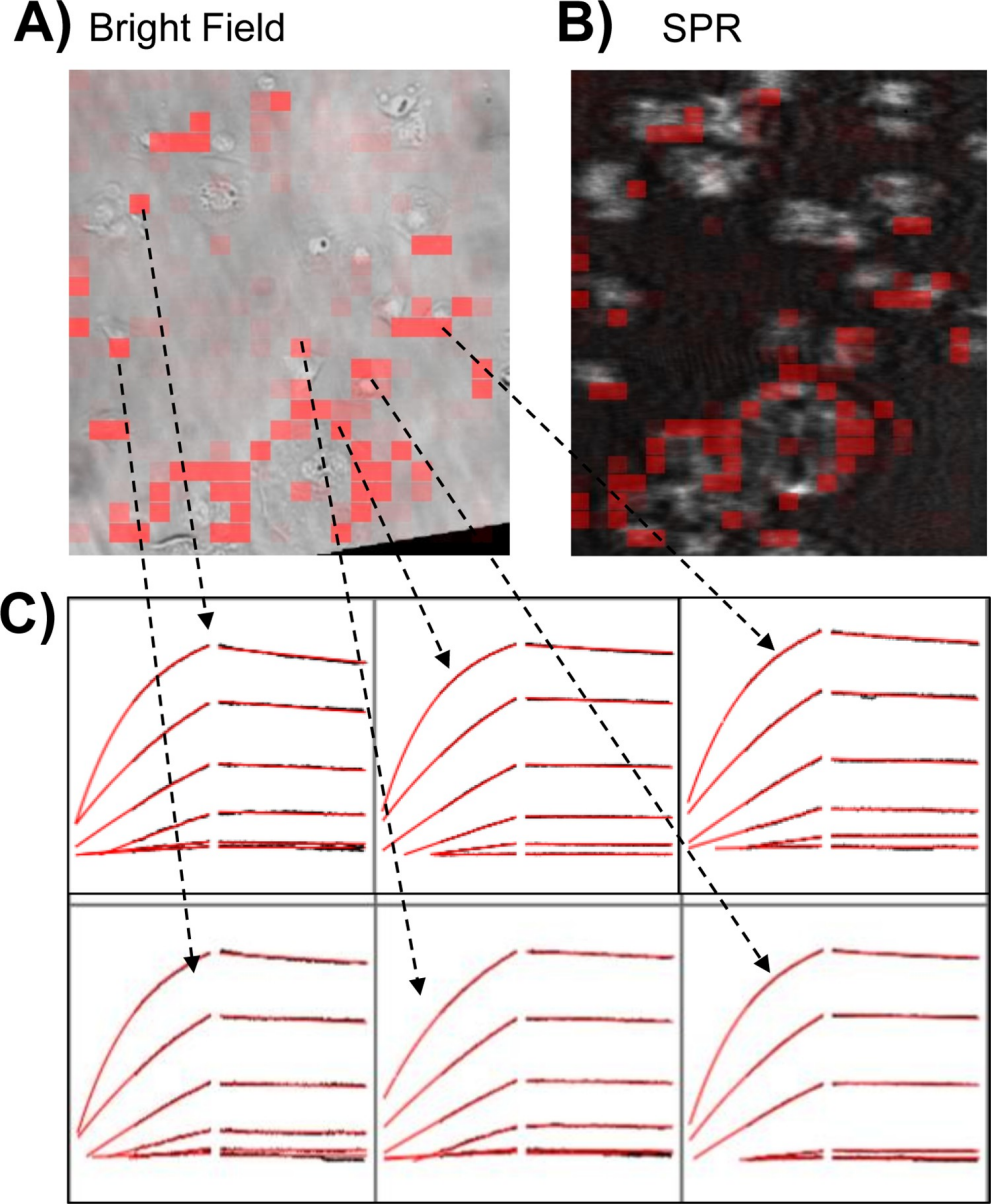
Con A lectin binding kinetics on BxPC3 pancreatic cancer cell surface. **A**) bright field image of BxPC3 pancreatic cancer cells. **B**) Corresponding SPR image. Overlaid red highlights indicating areas of detected Con A binding interaction, which overlap very closely with the cell regions and indicate high specificity. **C**) A small sampling of sensorgrams taken from hundreds responsive ROIs is shown. Arrows from cells point to their respective binding response sensorgrams. Serial injections of Con A lectin solutions (0.102, 0.307, 0.923, 2.77, 8.33, 25, and 75 Nm) were exposed to the cells. Sensorgrams were fitted using ImageSPR software.

BxPC3 pancreatic cancer cells were seeded on sensor chips coated with collagen (see materials and methods) and mounted on the SPRm 200 instrument for cell-based kinetic analysis evaluation (**Fig 1**). N- and O-linked glycan binding kinetics in BxPC3 cells were profiled with Con A and HPA lectins, respectively, and Mucin-4 binding interactions were profiled with the anti-MUC-4 monoclonal antibody, which targets the extracellular region of MUC-4 receptor. Analysis of the on-cell binding kinetics are a result of hundreds of SPR response curves directly measured from each cell on the sensor chip (see **Fig 2, S1 and S2 Figs**). To do this, SPR images of the cell-seeded sensor chip were collected during the sample exposure and rinse processes. Every responsive area was fit with a classical 1:1 or 1:2 kinetic binding interaction model to extract kinetic parameters, and was highlighted by a red square, mapping it as a binding region of interest (ROI). In the case of Con A and anti-MUC4, a 1:1 kinetic interaction model best described the observed binding responses (**Fig 2 and S2 Fig**). Secondary influences were insufficient to warrant the need for compensation with a 1:2 model. However, in the case of HPA binding interactions, secondary influences were sufficiently high to warrant the need for compensation with a 1:2 kinetic interaction model (**S1 Fig**). Application of the 1:2 kinetic model for HPA interactions is consistent with the various arrangements of O-GalNAc glycans (HPA epitopes), also known as mucin-type O-glycans, which are highly localized to only certain types of membrane proteins [55, 56]. This makes the O-glycan observed interactions susceptible to crowding and kinetic interference, unlike N-linked glycans which are more delocalized and scattered across multiple different types of membrane glycoproteins [57]. Non-specific binding was determined by comparing the number of ROIs found on bare areas or areas with no cells relative to ROI areas with cells. Minimal non-specific binding was observed for all anti-MUC-4 and lectins interactions (**Fig 2B and S1B, S2B Figs**). Additionally, total binding response from bare and cell areas was determined, and no response was observed from bare areas relative to cell areas (**S3 Fig**). Every binding interaction study was repeated five times.

### PNGase F enzymatic deglycosylation of BxPC3 cells elucidates Con A binding heterogeneity

**Fig 3:**
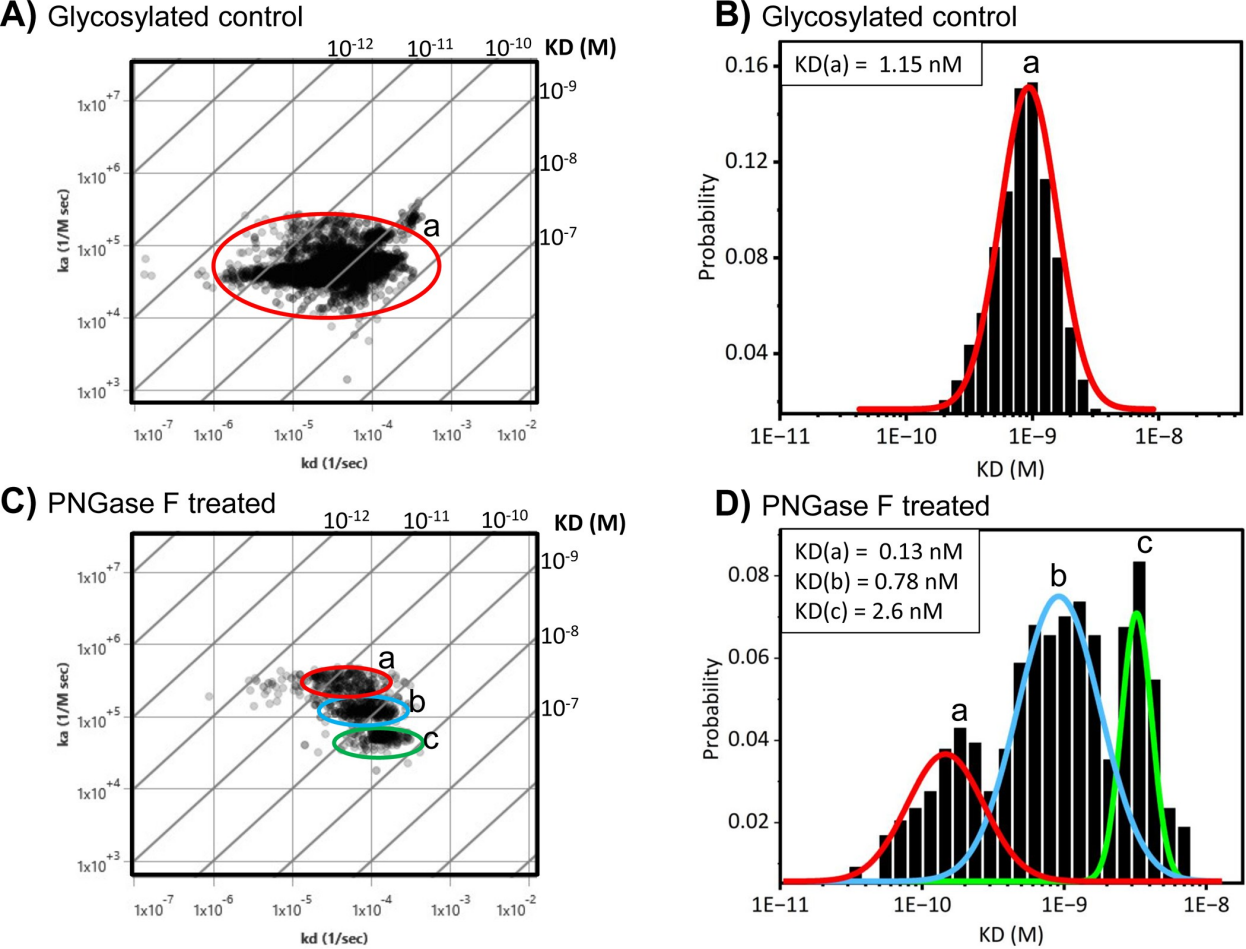
Con A binding affinity and heterogeneity increases upon enzymatic N-linked deglycosylation of BxPC3 cells. **A**) Affinity isotherm plot extracted from hundreds of responsive ROIs showing Con A binding population on glycosylated BxPC3 cells. **B**) Histogram describing total statistic kinetic interactions and distributions for Con A on glycosylated BxPC3 cells. **C**) Affinity isotherm plot extracted from hundreds of responsive ROIs for Con A on deglycosylated BxPC3 cells. **D**) Histogram describing total statistic of the kinetic interactions and distributions for Con A on deglycosylated BxPC3 cells. Diagonal lines of isoaffinity plots represent KD. *a*, *b*, and *c* represent binding interaction modes. Histogram data were graphed and fitted using Origin 2023b. Affinity isotherm plots were acquired using ImageSPR software.

It has long been recognized that aberrant N-linked glycosylation is a hallmark of most types of cancer, and it is associated with tumor development as well [7, 13, 17, 57–59]. Con A is one of the most widely used lectins in biomedical research, which has also been used in cancer biomarker discovery [60]. Additionally, this lectin has been used to verify enzymatic deglycosylation of N-linked glycans with PNGase F in whole cells in the past [36, 61]. Therefore, Con A was implemented in this study with SPRM to determine N-linked glycan binding kinetic changes upon enzymatic deglycosylation with PNGase F, which specifically removes N-linked glycans [62], in pancreatic cancer cells. To accomplish this, BxPC3 pancreatic cancer cells were treated with PNGase F enzyme to remove N-glycans. End-point analysis data with fluorescence microscopy and SPRM response data revealed a significant decrease (~ 70%) in Con A binding upon enzymatic deglycosylation with PNGase F (**S4 and S5 Figs**). Next, the kinetic parameters extracted from every responsive Con A binding ROI were aggregated to form affinity isotherm plots and histograms (**Fig 3**). To quantify the kinetic parameters of the predominant binding modes, gaussian distributions were fit to histograms of the kinetic interactions such that the mean values of the distributions revealed the predominate values for the equilibrium dissociation (KD), association rate (*k*_*a*_), and dissociation rate (*k*_*d*_) constants. A single predominant binding mode for Con A in glycosylated control BxPC3 cells was observed as determined by the affinity isotherm plot and histogram (**Fig 3A and 3B**), producing an equilibrium dissociation constant (K_D_) of 1.15 nM (95% CI of 1.13 to 1.17 nM), an association rate constant (*k*_*a*_) of 4.7X10^4^ M^-1^ s^-1^, and a dissociation rate constant (*k*_*d*_) of 4.3X10^-5^ s^-1^ (**Fig 3B and Table 1**). However, after BxPC3 cells underwent PNGase F treatment to remove N-linked glycans, three distinct binding interaction modes for Con A on BxPC3 cells were observed, as shown in the isoaffinity plot and histogram (**Fig 3C and 3D**). Three KDs were measured at 0.13 nM (95% CI of 0.13 to 0.14 nM), 0.78 nM (95% CI of 0.74 to 0.83 nM), and 2.6 nM (95% CI of 2.52 to 2.64 nM) on deglycosylated cells for Con A (**Fig 3D and Table 1**). Three association rate constants at 2.9X10^5^ M^-1^ s^-1^, 1.2X10^5^ M^-1^ s^-1^, and 5.3X10^4^ M^-1^ s^-1^, and three dissociation rate constants at 4.5X10^-5^ s^-1^, 8.6X10^-5^ s^-1^, and 1.3X10^-4^ s^-1^ (**Table 1**).

**Table 1:** Kinetic parameters of all lectin and antibody interactions.

Cell	Analyte	KD (nM)	CI (95%)	ka (M^-1^s^-1^)	kd (s^-1^)
**Glycosylated BxPC3 Cells (Control)**	**Con A**	a) 1.15	a) 1.13 to 1.17	a) 4.75X10^4^	a) 4.3X10^-5^
	**Anti-MUC-4**	a) 3.7 b) 14.5	a) 3.2 to 4.1 b) 13 to 15	a) 4.3X10^5^ b) 1.2X10^5^	a) 1.5X10^-3^ b) 1.4X10^-3^
	**HPA**	a) 1.2 b) 59 c) 0.82 d) 0.022	a) 1.1 to 1.3 b) 51 to 60 c) 0.77 to 0.84 d) 0.019 to 0.02	a) 6.1X10^6^ b) 2.0X10^5^ c) 4.3X10^5^ d) 7.0X10^6^	a) 7.4X10^-3^ b) 1.0X10^-2^ c) 3.5X10^-4^ d) 1.5X10^-4^
**Deglycosylated BxPC3 Cells**	**Con A**	a) 0.13 b) 0.78 c) 2.6	a) 0.13 to 0.14 b) 0.74 to 0.83 c) 2.52 to 2.64	a) 2.9X10^5^ b) 1.2X10^5^ c) 5.3X10^4^	a) 4.5X10^-5^ b) 8.6X10^-5^ c) 1.3X10^-4^
	**Anti-MUC-4**	a) 0.099 b) 1.5	a) 0.82 to 0.10 b) 1.3 to 2.4	a) 1.1X10^7^ b) 7.0X10^5^	a) 1.0X10^-3^ b) 1.1X10^-3^
	**HPA**	a) 0.58 b) 10 c) 0.25 e) 4.5	a) 0.51 to 0.61 b) 9 to 11 c) 0.22 to 0.26 e) 4.3 to 5.0	a) 6.2X10^5^ b) 9.2X10^5^ c) 2.0X10^7^ e) 2.4X10^4^	a) 3.6X10^-4^ b) 1.0X10^-2^ c) 2.5X10^-3^ e) 1.1X10^-4^

A 1:1 binding model was applied to the analysis of all Con A kinetic data. The sub-cellular resolution of SPRM enables the usage of affinity isotherm plots to provide a uniquely informative representation of binding heterogeneity by revealing predominant modes of kinetic binding interactions that would otherwise be hidden with traditional endpoint and affinity measurement techniques. The predominant binding modes are a consequence of various kinetic binding interactions resulting from cellular heterogeneity that are observed most frequently in the population. It has been reported by other studies that there are three predominant glycoforms that Con A binds to: high affinity modes for the core mannose in both oligomannose-type N-glycans and hybrid-type N-glycans, and a weak affinity mode for mannose in the complex type bi-antennary N-glycans [61, 63, 64], Con A does not bind to mannose in complex tri- or tetra antennary N-glycans [65–68]. Our results show that upon partial removal of N-linked glycans, the various binding interaction modes for Con A can be better differentiated, thereby enabling the unique capability of SPRM to quantify the heterogeneity and interaction kinetics as a result of these predominant interaction modes. These results highlight the complexity of glycan diversity on pancreatic cancer cells, which can range from complex type N-glycans containing bi-, tri, and tetra-antennary branches, oligomannose type N-glycans, and hybrid type N-glycans [12, 57]. While the results do not attempt at identifying the specific glycoforms being recognized by Con A, the binding kinetics of three new interaction modes for Con A were identified by SPRM. This level of resolution on binding heterogeneity using kinetic analysis would not be readily observable using traditional end point analysis or other low-resolution approaches that average the binding responses over multiple cells.

### Enzymatic N-linked deglycosylation enhances Mucin-4 binding affinity

**Fig 4:**
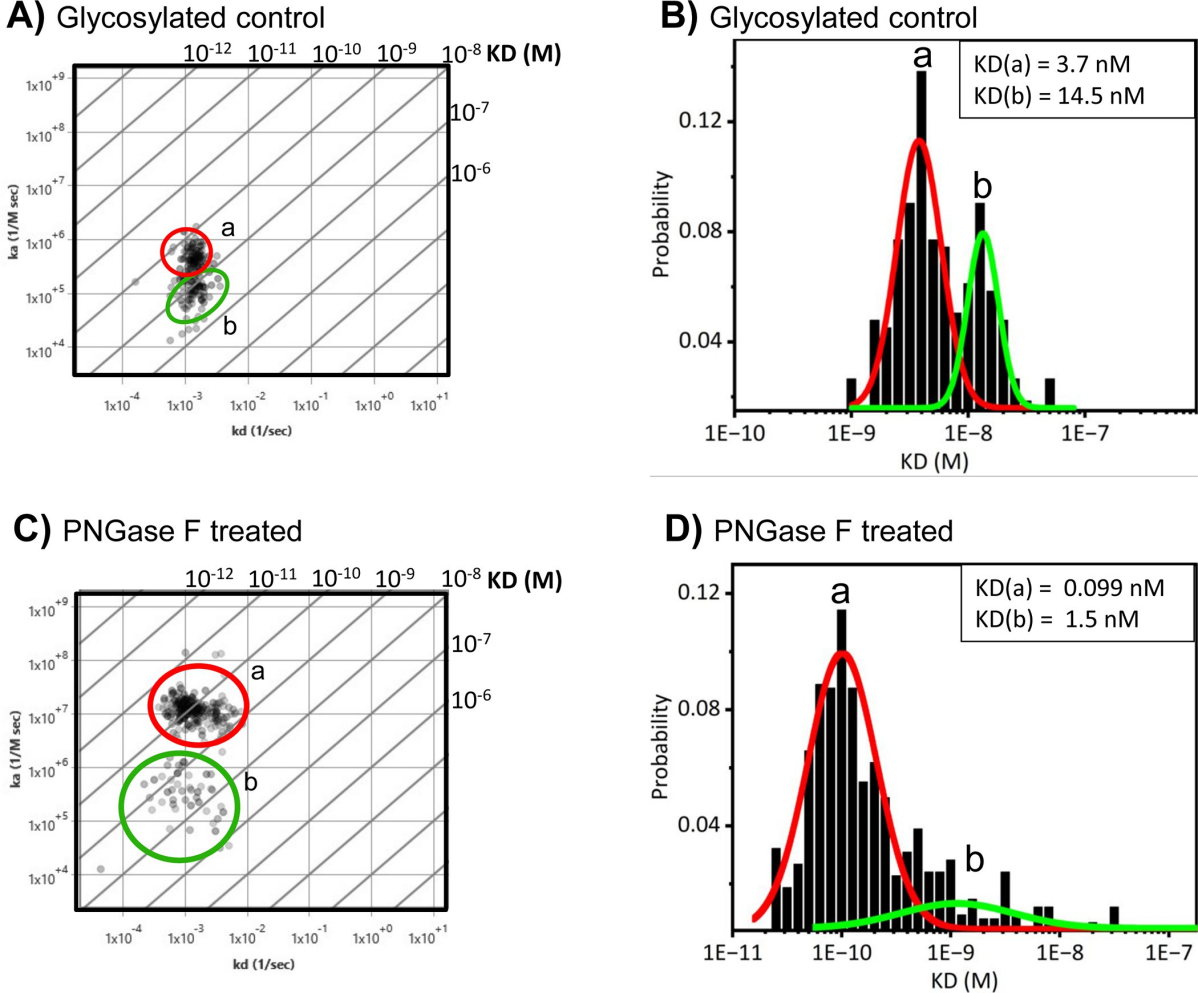
Binding affinity of Anti-MUC4 monoclonal antibody increases on deglycosylated BxPC3 pancreatic cancer cells. **A**) Affinity isotherm plot extracted from hundreds of responsive ROIs for anti-MUC-4 on glycosylated BxPC3 cells which produces two distinct binding interactions, labeled as modes *a* and *b*. **B**) Histograms describing total statistic kinetic interactions and distributions for anti-MUC-4 on glycosylated BxPC3 cells. **C**) Affinity isotherm plot extracted from hundreds of responsive ROIs for anti-MUC-4 on deglycosylated BxPC3 cells which produces higher affinity and faster association for the two binding interaction modes labeled as modes *a a*nd *b*. **D**) Histograms describing total statistic kinetic interactions and distributions for anti-MUC-4 on N-linked deglycosylated BxPC3 cells. *a* and *b* represent binding interaction modes. Histogram data were graphed and fitted using Origin 2023b. Affinity isotherm plots were acquired using Image SPR software.

To further investigate the influence of N-linked glycosylation on Mucin-4 within the cellular environment, the binding interactions of monoclonal antibody anti-MUC-4, which targets the extracellular region of MUC-4 (R&D Sytems), was studied with SPRM. MUC-4 is predominantly expressed in pancreatic cancer cells, and it has been identified as an ideal vaccine candidate for PC [22]. Therefore, an accurate understanding of MUC-4 binding interactions within the aberrant N-linked glycosylated cellular environment is key to the development of new vaccines and drug therapies. Our results reveal two distinct anti-MUC-4 binding interaction modes in glycosylated BxPC3 cells (**Fig 4A and 4B**), with mode (*a)* having a 4x faster on-rate (4.3X10^5^ M^-1^s^-1^ with a 95% CI of 4.1 to 5.0 M^-1^s^-1^) and higher affinity (3.7 nM with a 95% CI of 3.2 to 4.1 nM) and being much more predominant than the other mode *(b)*, which had a k_a_ of 1.2X10^5^ M^-1^s^-1^ with a 95% CI of 1.1X10^5^ to 1.3X10^5^, and a KD of 14.5 nM with a 95% CI of 13 to 15 nM. Data are representative from 5 different experiments. From every single experiment kinetic data was collected across hundreds of sensorgrams directly derived from single cells.

The extracellular domain of MUC-4 is exclusively O-linked glycosylated [25, 26], making the presence of any N-linked glycoforms in this region the likely result of encroachment from neighboring glycoproteins [54, 62]. Upon partial removal of N-linked glycans with PNGase F enzyme in PC cells, two distinct binding interaction modes are still observed. However, mode (*a*) shifted to ~25x faster on-rate (1.1X10^7^ M^-1^s^-1^ with a 95% CI of 1.0X10^7^ to 1.2X10^7^ M^-1^s^-1^) and 37x higher affinity (0.099 nM with a 95% CI of 0.82 to 0.10 nM) relative to the glycosylated control mode (*a*). Mode (*b*) displayed a much broader distribution with a low probability, and a ~9x higher affinity at 1.5 nM with a 95% CI of 1.3 to 2.4 nM, relative to the glycosylated control mode (*b*) (**Fig 4C and 4D, Table 1**). All data are representative from 5 different experiments. A 1:1 binding model was applied to all MUC-4 data analysis. We attribute the second binding mode *b* to a hindered mode. This is supported by the observation that after partial N-linked glycan deglycosylation, the binding occurrences of mode *b* are significantly diminished, whereas those of mode *a* are significantly more abundant. These results reveal the complex influence of extraneous N-linked glycans on the binding heterogeneity of MUC-4 of pancreatic cancer cells, which renders important implications to improve MUC-4 detection and its utilization as a potential biomarker of PC.

### N-linked glycan deglycosylation unmasks new HPA O-linked glycan heterogeneity

**Fig 5:**
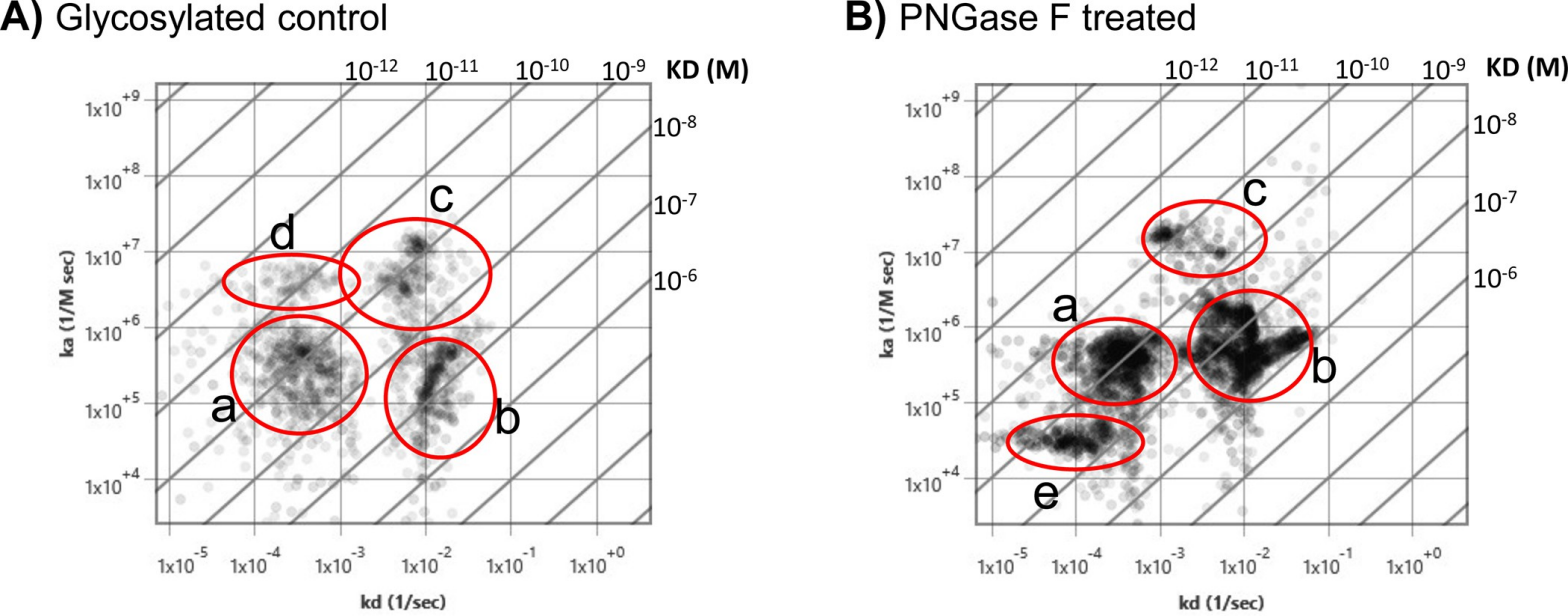
Removal of N-linked glycans unmasks HPA binding heterogeneity. **A**) Affinity isotherm plot extracted from hundreds of responsive ROIs showing four distinct HPA binding population modes on glycosylated BxPC3 cells. **B**) Affinity isotherm plot extracted from hundreds of responsive ROIs for HPA on deglycosylated BxPC3 cells showing four distinct binding populations. Mode e highlights the appearance of a new binding population mode upon enzymatic deglycosylation. *a*, *b*, *c*, *d*, and *e* represent binding interaction modes. Affinity isotherm plots were acquired using ImageSPR software.

Mucins are highly rich with O-linked glycosylated tandem repeats [69], and they are the main membrane proteins carrying O-glycans [55]. Here we further investigated the influence of N-linked glycosylation on O-glycan binding kinetic interactions. The lectin Helix pomatia agglutinin was used to profile the O-GalNAc glycans, which are also known as mucin-type O-glycans [55, 56]. This lectin can recognize multiple O-glycan epitopes and is widely used in cancer biomarker discovery [70–72]. SPRM results identified three predominant binding interaction modes for HPA lectin in glycosylated BxPC3 cells, which produced KDs of 1.2 nM (95% CI of 1.1 to 1.3 nM) (mode *a*), 59 nM (95% CI of 51 to 60 nM) (mode *b)*, and 0.82 nM (95% CI of 0.77 to 0.84 nM) (mode *c*) (**Fig 5A**). A faint fourth binding mode (mode *d*) is observed at around 0.022 nM (95% CI of 0.019 to 0.023), but it is not as abundant as the other three modes with a low probability of 0.014 (**Fig 5A**). A 1:2 binding model was applied to process the data, helping to compensate for secondary influences. By varying both the sample concentration and measurement regions, heterogeneity of the HPA interaction on PC cells could be identified. After cells underwent PNGase F treatment under non-denaturing conditions to remove N-linked glycans, a fourth mode (*e*) of binding interaction is clearly presented, having a significantly lower on-rate (2.4X10^4^ M^-1^s^-1^ with a 95% CI of 2.3 to 3.1) and weaker affinity (4.5 nM with a 95% CI of 4.3 to 5.0 nM) than modes *a* and *c* and (**Fig 5B**), possibly indicating that this mode is particularly sensitive to steric hinderance or may still be significantly hindered. Modes *a*, *b*, and *c* shifted to slighted higher affinities at 0.58 nM (95% CI of 0.51 to 0.61), 10 nM (95% CI of 9 to 11 nM), and 0.25 nM (95% CI of 0.22 to 0.26), respectively. Interestingly, the faint mode *d* is no longer present (**Fig 5**). Data are representative from 5 different experiments. From every single experiment, kinetic data was collected across hundreds of sensorgrams directly derived from single cells. These results suggest that N-linked glycosylation significantly influences the HPA interaction by not only hindering access but also completely inhibiting one of the binding modes (mode *e*), which becomes more readily accessible after N-linked deglycosylation. Clearly, HPA appears to have multiple presentations of its GalNAc epitopes for binding interactions. Although the identity of the specific glycoforms HPA recognizes are yet unknown, we reveal here the binding kinetics of the most prominent HPA epitopes under fully glycosylated and partial deglycosylated conditions.

## Discussion

The objective of this work was to investigate the influence of glycans on Mucin-4 (MUC-4) binding interactions in pancreatic cancer (PC) cells, and to determine glycan heterogeneity (as defined here) with Con A and HPA lectins upon removal of N-linked glycans. This was achieved using SPRM technology, which is an ideal technique for studying the affinity and kinetics of membrane receptors in their native environment. The results from our experiments revealed the following trends; N-linked deglycosylation of BxPC3 cells 1) exposes Con A binding heterogeneity, 2) enhances MUC-4 binding affinity, and 3) unmasks new HPA O-linked glycan binding modes. This study has identified multiple aspects of cancer cell membrane glycoprotein binding kinetics and heterogeneity ranging from N- and O-linked glycan kinetics and antibody binding kinetics, and the discovery of a new role of N-linked glycosylation in MUC-4 binding interactions.

Heterogeneity as described here encompasses all the different types of binding interactions resulting from receptor hindrance and variations in receptor glycoforms (e.g., glycosylated vs deglycosylated). SPRM revealed several unique aspects of N-linked glycosylation heterogeneity resulting from different glycoforms. It was found that Con A affinity and heterogeneity increased in the PNGase F treated cells (**Fig 3**). Although fluorescence results confirmed a significant reduction in Con A staining upon deglycosylation, SPRM identified more detailed information on binding interactions. After enzymatic deglycosylation, the affinity isotherm plots and histograms confirmed the presence of three binding interaction modes in Con A binding heterogeneity, which cannot be readily identified by end-point analysis alone. Here, the binding heterogeneity is likely attributed to the complex N-linked glycoform diversity found within cancer cells, which results in several distinct interaction modes after removal of N-linked glycans. This may be a consequence of PNGase F inability to cleave certain specific N-linked glycoforms, such as N-glycans with an alpha 1-6-linked fucose attached to the reducing end of GlcNAc [73], thus leaving behind the remaining interaction modes. Additionally, Con A is a multivalent lectin with more than one glycan binding site [74], hence it’s likely that other sugar epitopes become more apparent after removal of N-linked glycans. It has been reported by other studies that there are three predominant glycoforms that Con A binds to: high affinity modes for the core mannose in both oligomannose-type N-glycans and hybrid-type N-glycans, and a weak affinity mode for mannose in the complex type bi-antennary N-glycans [61, 63–65]. However, more work is required to identify which specific glycoforms contribute to the binding heterogeneity observed.

In this study, it was discovered that cell surface N-linked glycosylation strongly hinders the interactions of the anti-MUC4 monoclonal antibody that targets the extracellular region of MUC-4 receptor (**Fig 4**). However, since the MUC-4 extracellular region is mainly O-linked glycosylated [18], N-linked glycoforms from neighboring membrane glycoproteins are attributed here to greatly influencing the accessibility to the MUC-4 receptor. By enzymatically removing most of the N-linked glycans from membrane glycoproteins in BxPC3 pancreatic cancer cells, the increased MUC-4 antigen exposure resulted in ~25x faster association, ~37x higher affinity, and two distinct well-resolved interaction modes. We attribute the second binding mode *b* to a hindered mode. This is supported by the observation that after N-linked glycan deglycosylation, the binding occurrences of mode *b* are significantly diminished, whereas those of mode *a* are significantly more abundant. The implications of these results suggest that N-linked deglycosylation has the potential to greatly improve MUC-4 detection and its potential utilization as a diagnostic biomarker in PC.

In addition, the influence of N-linked glycosylation on HPA binding interactions, which is an O-linked glycan lectin, was investigated in this study (**Fig 5**). While the role of O-glycans on MUC-4 interactions was not determined, it was discovered that N-linked glycosylation generally hinders O-linked glycan binding interaction. This study identified a hidden HPA binding interaction mode with a characteristically slow association rate constant at 3.7X10^4^ M^-1^s^-1^ that becomes more accessible to lectin binding upon removal of N-linked glycans in BxPC3 cells. HPA was initially believed to bind only to N-acetelyllactosamine residues or the Tn antigen on cancer cells [75], but it appears that this lectin has a much broader selection of O-linked glycan epitopes [72]. For instance, based on other reports, there appears to be three predominant cell surface HPA epitopes; GalNAcα1,3Gal, β-GalNAc, and GlcNAcβ1,4Gal [72]. Some studies have shown that HPA also has an affinity for the intracellular modification O-GlcNAc [72], as well as for monosialylated oligosaccharides in breast cancer [70]. In this study, a total of four binding interactions modes upon removal of N-linked glycans became more apparent relative to the glycosylated control, and their binding kinetics were determined. Additionally, this study identified a faint mode (mode *d)* with high affinity that interestingly disappears after deglycosylation, possibly inferring that N-linked glycans might also play a limited role in enhancing some modes of HPA interactions.

This is the first study to implement SPRM to characterize the influence of the human glycocalyx within pancreatic cancer cells on binding interactions of the novel PC vaccine candidate MUC-4. *In vitro w*hole-cell binding kinetics without disrupting the native environment of membrane glycoproteins is critical to revealing more biologically relevant binding interaction information. Although there are various technologies, such as Quartz Crystal Microbalance (QCM) [45, 76–79] and Ligand Tracer (LT) [80, 81] that can also reveal glycan binding kinetics in whole cells, such technologies do not have the sub-cellular resolution of SPRM that enables detailed statistical studies of binding heterogeneity [4, 50–53]. Aberrant glycosylation is a well-established hallmark of cancer [10–12, 82–91], and aberrant N-glycosylation has been studied extensively and shown to play a major role in binding interactions of critical therapeutic targets [7, 8, 35, 45]. Hence, testing methods that consider aberrant glycosylation heterogeneity for measuring binding kinetics of new therapeutic drugs should be established.

## Supporting information

S1 Fig HPA lectin binding kinetics on BxPC3 pancreatic cancer cell surface. **A**) bright field image of BxPC3 pancreatic cancer cells with overlaid red highlights indicating areas of detected HPA binding interactions. **B**) Corresponding SPR image with overlaid red highlights indicating areas of detected HPA binding interaction, which overlap very closely with the cell regions and indicate high specificity. **C**) A small sampling of sensorgrams taken from hundreds of responsive ROIs is shown. Arrows from cells point to their respective binding response sensorgrams. Serial injections of HPA lectin solutions (0.102, 0.307, 0.923, 2.77, 8.33, 25, and 75 nM) were exposed to the cells. Sensorgrams were fitted using Image SPR software.
(PDF)

S2 Fig Anti-MUC4 binding kinetics on BxPC3 pancreatic cancer cell surface. **A)** bright field image of BxPC3 pancreatic cancer cells with overlaid red highlights or ROIs indicating areas of detected Anti-MUC4 binding interaction on cells, which overlap very closely with the cell regions and indicate high cell specificity. **B)** Corresponding SPR image with overlaid red highlights indicating Anti-MUC4 binding response areas **C)** A small sampling of sensorgrams taken from hundreds of responsive ROIs directly derived from cells is shown. Serial injections of anti-MUC-4 antibody solutions (0.390, 0.781, 1.56, 3.12, 6.25, 12.5, and 25 nM) were exposed to the cells. Sensorgrams were fitted using Image SPR software.
(PDF)

S3 Fig Representative data of sum average binding response from cell and bare areas in sensor chip with BxPC3 cells. **A**) Sum of average binding response obtained from cell areas. **B**) Sum of average binding response from bare areas.
(PDF)

S4 Fig Fluorescence microscopy reveals a significant reduction in N-glycans upon PNGase F treatment in BXPC3 pancreatic cancer cells. 75 nM of Fluorescently labeled Con A was exposed to the cells for 5 minutes, and fluorescence was captured immediately after.
(PDF)

S5 Fig SPRM technology reveals a significant reduction in N-glycans upon PNGase F treatment in BXPC3 pancreatic cancer cells. Total overall cell response and corresponding fitted sensorgrams upon Con A binding on control (glycosylated BxPC3 cells) and PNGase F treated cells (deglycosylated BxPC3 cells). Bar graph shows sum of average binding response from multiple individual cells of each control and deglycosylated cells.
(PDF)
