# Supplementary figures and images for: Surface plasmon resonance microscopy identifies glycan heterogeneity in pancreatic cancer cells that influences mucin-4 binding interactions
Source: PLoS One. 2024 May 22;19(5):e0304154. doi: 10.1371/journal.pone.0304154 (PMC11111020; doi:10.1371/journal.pone.0304154)

**A)** Bright field

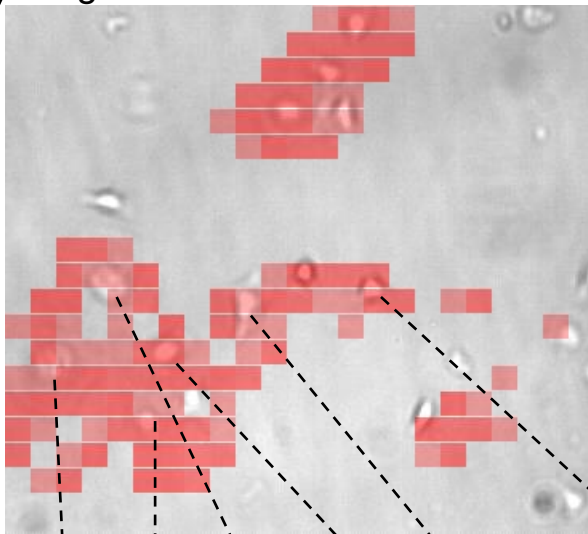

**B)** SPR

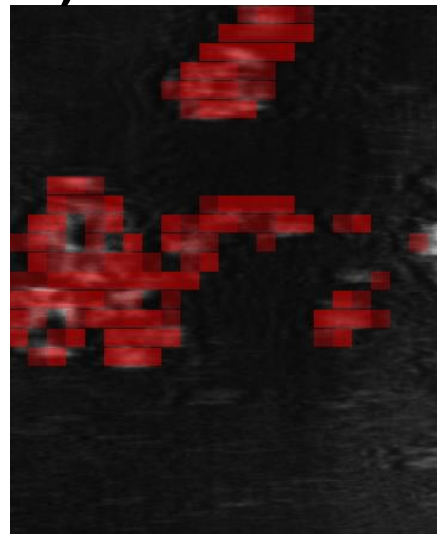

**C)**

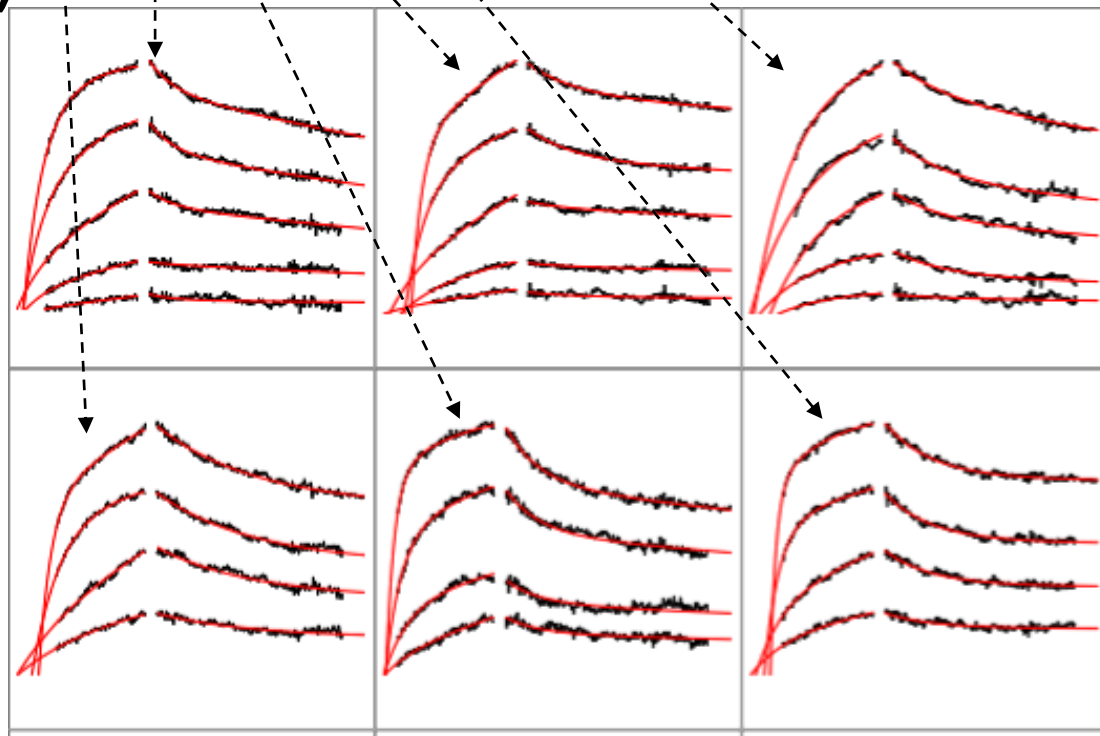

Supplement: S1 Fig — A) bright field image of BxPC3 pancreatic cancer cells with overlaid red highlights indicating areas of detected HPA binding interactions. B) Corresponding SPR image with overlaid red highlights indicating areas of detected HPA binding interaction, which overlap very closely with the cell regions and indicate high specificity. C) A small sampling of sensorgrams taken from hundreds of responsive ROIs is shown. Arrows from cells point to their respective binding response sensorgrams. Serial injections of HPA lectin solutions (0.102, 0.307, 0.923, 2.77, 8.33, 25, and 75 nM) were exposed to the cells. Sensorgrams were fitted using Image SPR software. (PDF) [file pone.0304154.s001.pdf]

**A)** Bright field

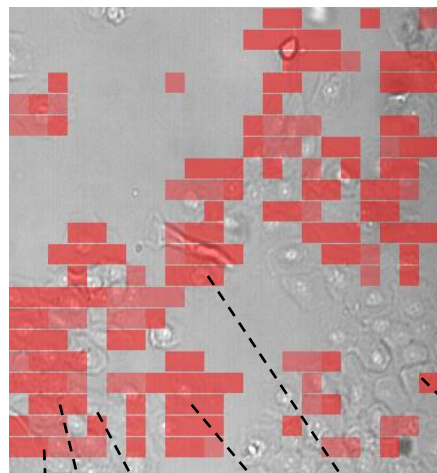

**B)** SPR

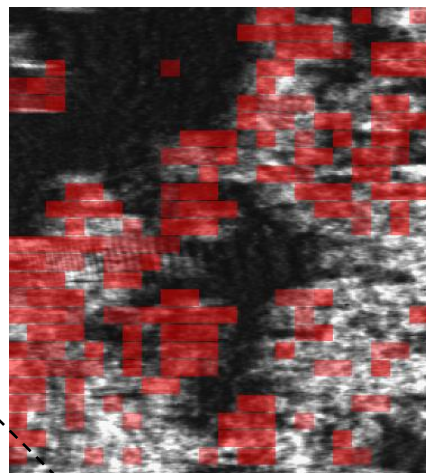

**C)**

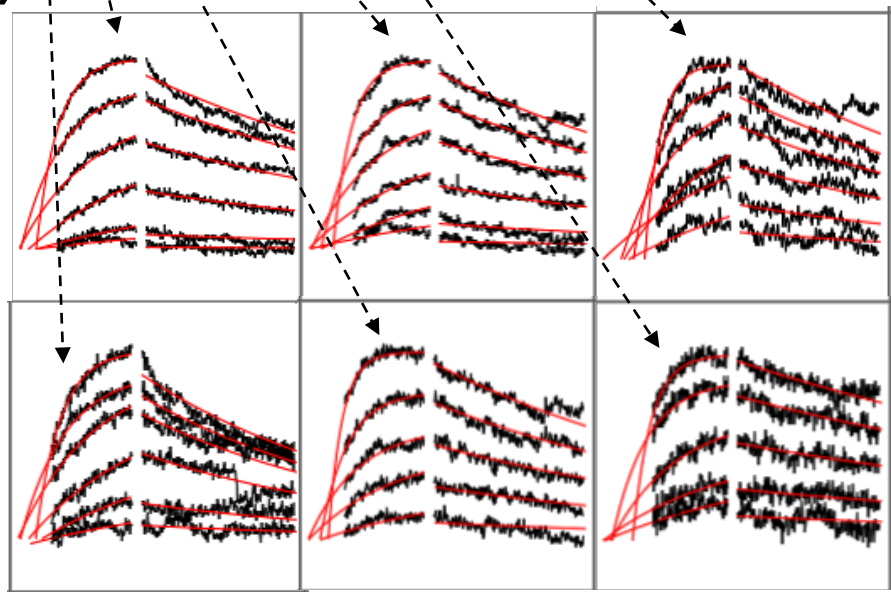

Supplement: S2 Fig — A) bright field image of BxPC3 pancreatic cancer cells with overlaid red highlights or ROIs indicating areas of detected Anti-MUC4 binding interaction on cells, which overlap very closely with the cell regions and indicate high cell specificity. B) Corresponding SPR image with overlaid red highlights indicating Anti-MUC4 binding response areas C) A small sampling of sensorgrams taken from hundreds of responsive ROIs directly derived from cells is shown. Serial injections of anti-MUC-4 antibody solutions (0.390, 0.781, 1.56, 3.12, 6.25, 12.5, and 25 nM) were exposed to the cells. Sensorgrams were fitted using Image SPR software. (PDF) [file pone.0304154.s002.pdf]

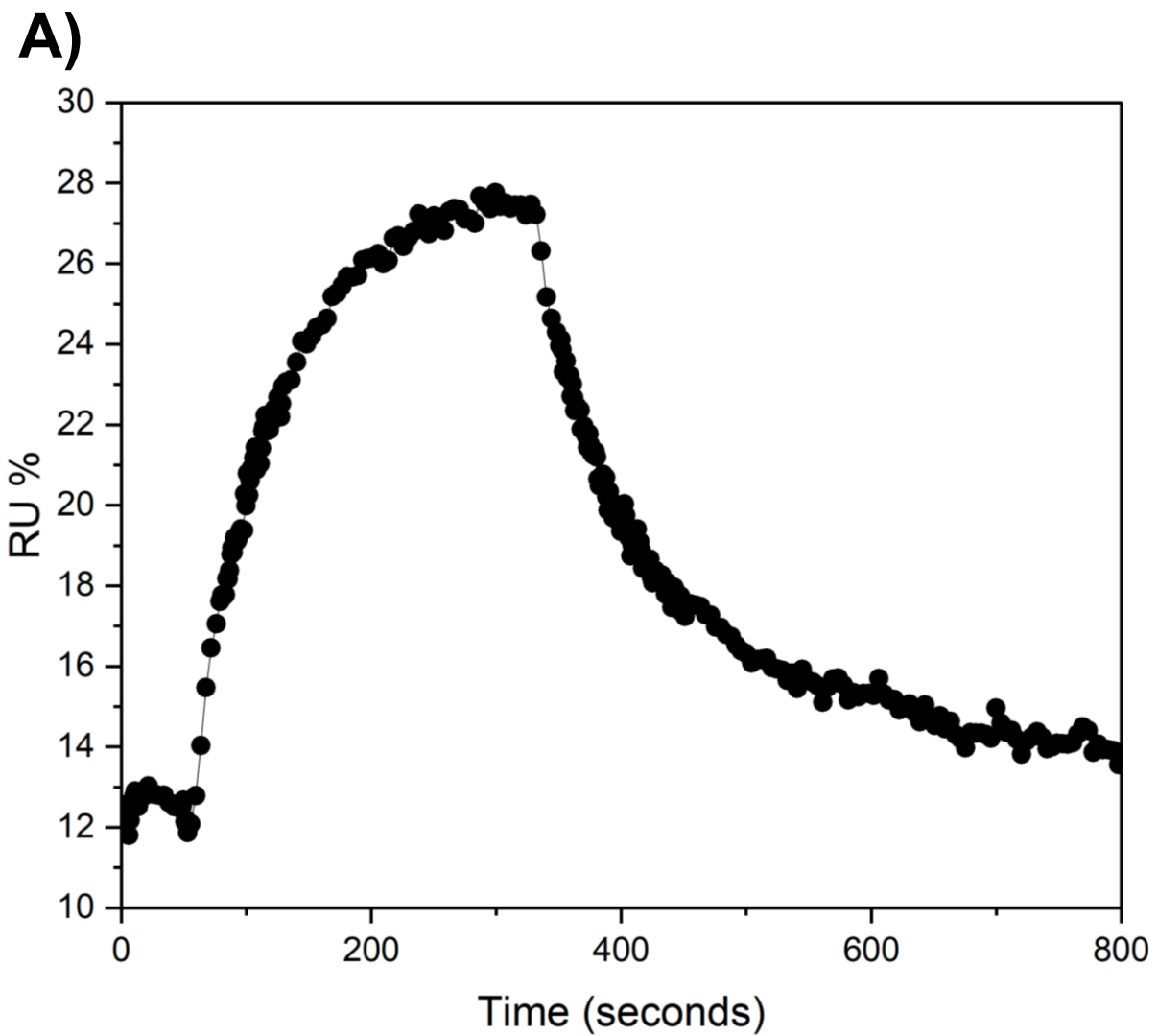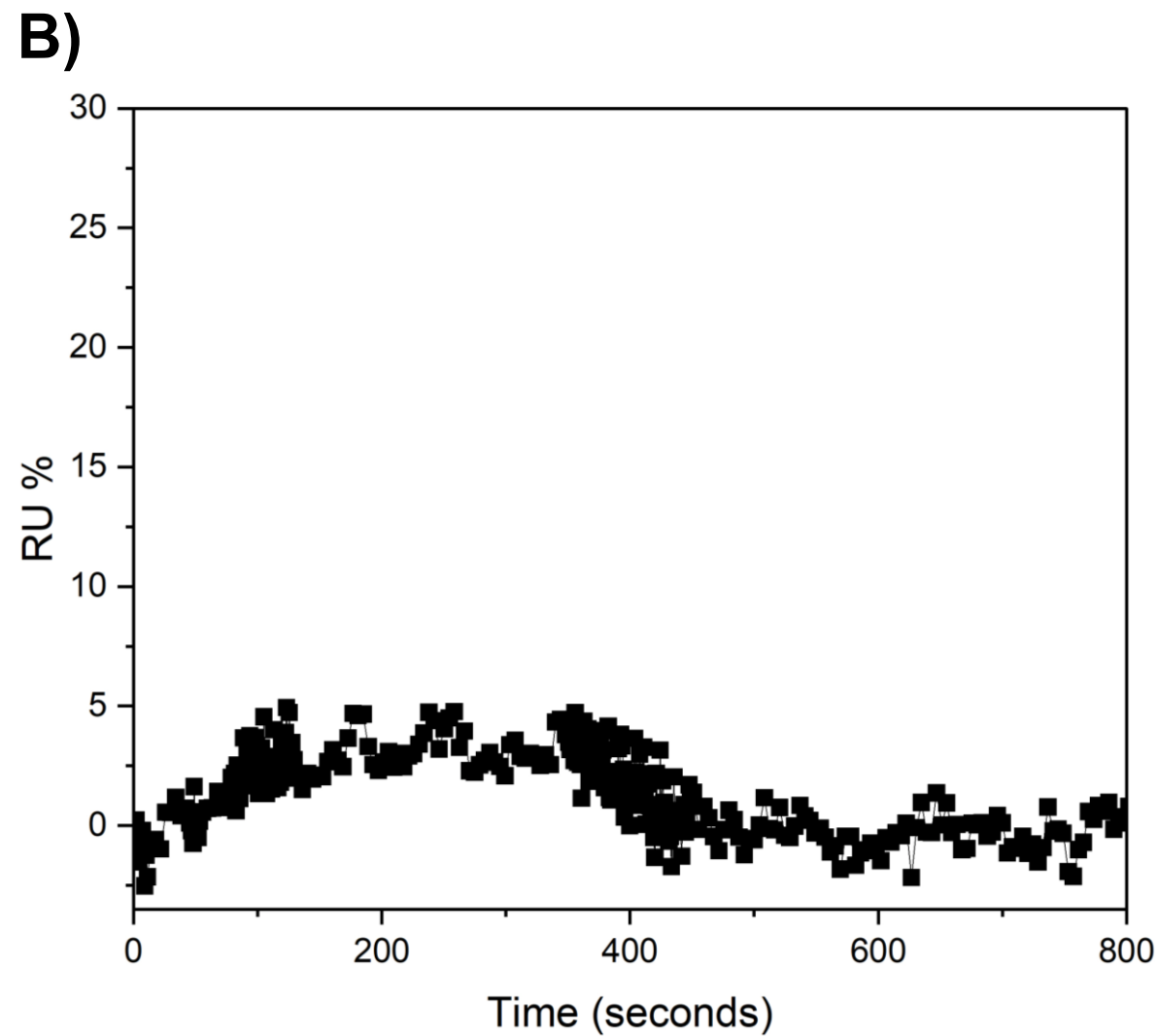

Supplement: S3 Fig — A) Sum of average binding response obtained from cell areas. B) Sum of average binding response from bare areas. (PDF) [file pone.0304154.s003.pdf]

### A) Glycosylated

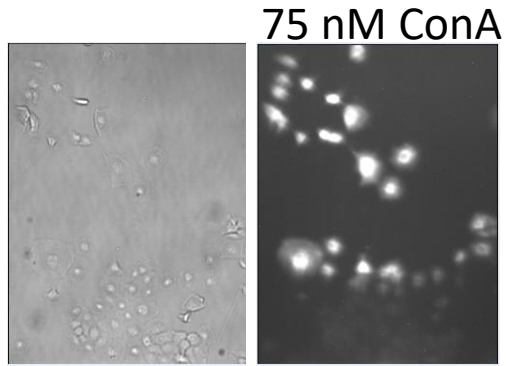

### B) Deglycosylated

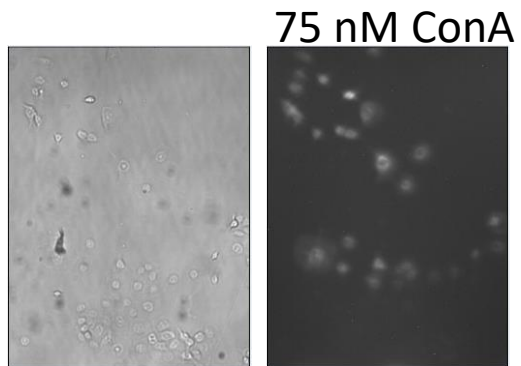

### Con A Response

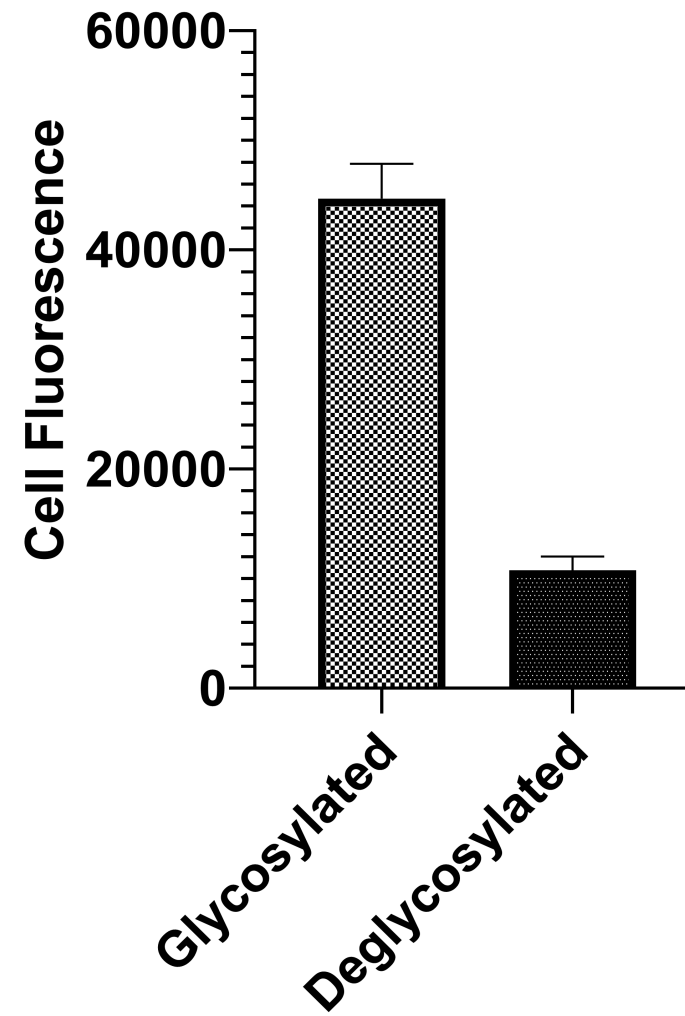

Supplement: S4 Fig — 75 nM of Fluorescently labeled Con A was exposed to the cells for 5 minutes, and fluorescence was captured immediately after. (PDF) [file pone.0304154.s004.pdf]
