# Supplementary material for: Surface plasmon resonance microscopy identifies glycan heterogeneity in pancreatic cancer cells that influences mucin-4 binding interactions
Source: PLoS One. 2024 May 22;19(5):e0304154. doi: 10.1371/journal.pone.0304154 (PMC11111020; doi:10.1371/journal.pone.0304154)

Con A Response

Glycosylated

Overall cell response (1143.22)

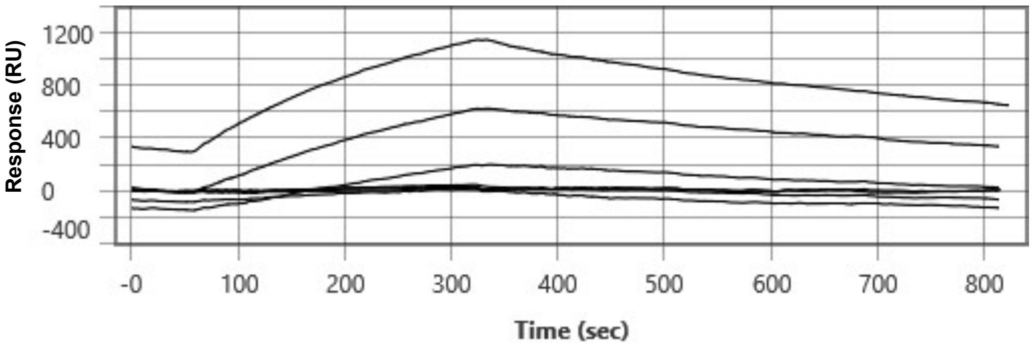

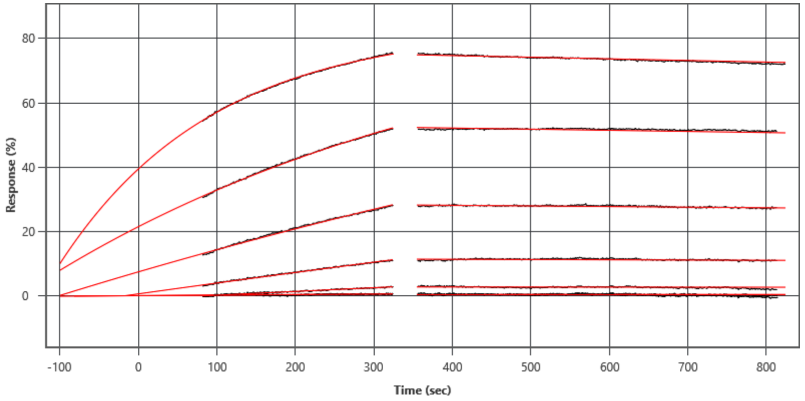

Deglycosylated

Overall cell response (306.67)

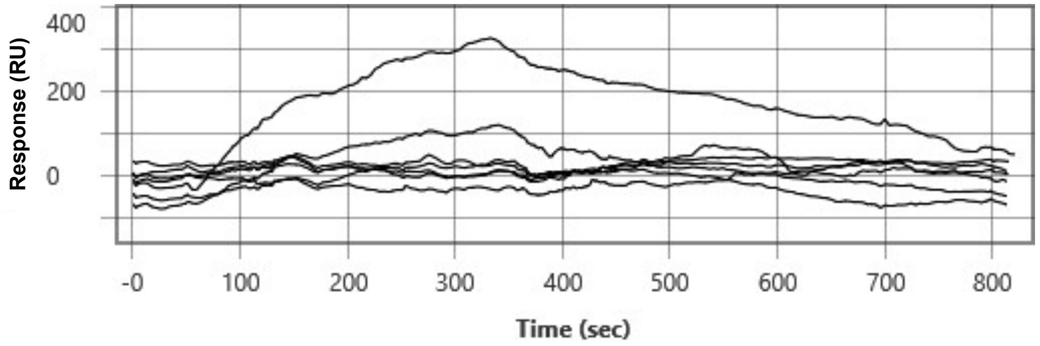

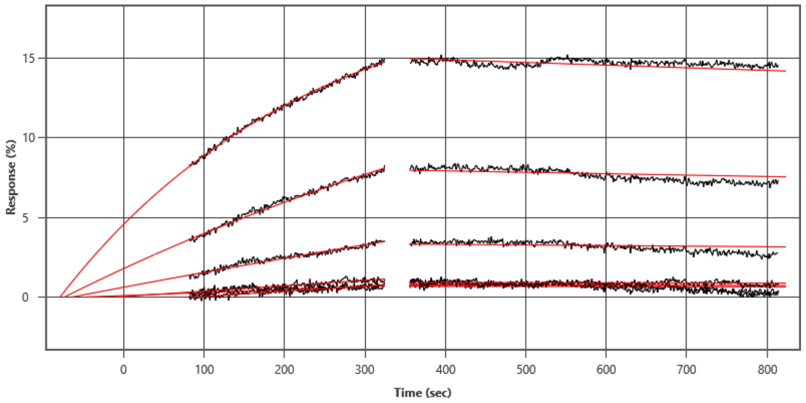

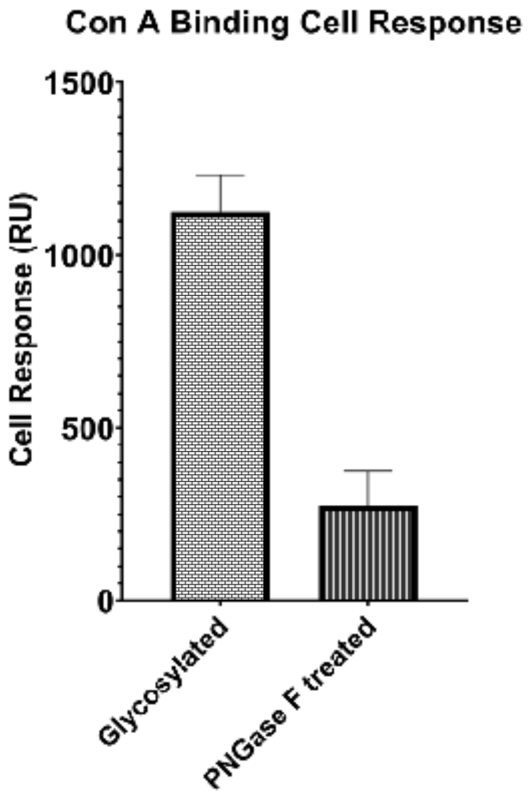

Supplement: S5 Fig — Total overall cell response and corresponding fitted sensorgrams upon Con A binding on control (glycosylated BxPC3 cells) and PNGase F treated cells (deglycosylated BxPC3 cells). Bar graph shows sum of average binding response from multiple individual cells of each control and deglycosylated cells. (PDF) [file pone.0304154.s005.pdf]
